# Physiological restorative benefits of different types of urban green spaces: a threshold model approach

**DOI:** 10.3389/fpubh.2026.1735199

**Published:** 2026-02-06

**Authors:** Danping Ma, Jianbin Lu, Qilong Wu, Jialin Zhang, Yueting Zhou, Dan Chen, Jingyi Hu, Shengyu He, Huacheng Zhao, Bo Yang

**Affiliations:** 1 School of Hydraulic Engineering, Zhejiang University of Water Resources and Electric Power, Hangzhou, China; 2 Zhejiang Key Laboratory of River-Lake Water Network Health Restoration, Hangzhou, China; 3 Wucheng District Forestry Pest Control and Quarantine Station, Jinhua, China; 4 School of Forestry and Biotechnology, Zhejiang A&F University, Hangzhou, China; 5 School of Innovation and Entrepreneurship, Zhejiang University of Water Resources and Electric Power, Hangzhou, China

**Keywords:** ecosystem service, physiological health, threshold model, urban green space, visual naturalness

## Abstract

**Introduction:**

As urbanization accelerates, understanding the specific health benefits of green spaces is crucial. This study investigates the physiological restorative effects of Urban Parks (UP), Remnant Forests (RF), and Wetland Parks (WP).

**Methods:**

We employed a novel physiological health effects (PHE) threshold model and EEG data to analyze participant responses to short-term exposure in these environments, establishing efficiency and benefit thresholds.

**Results:**

While all types provided benefits, they exhibited distinct patterns. UP demonstrated the most rapid and stable physiological stress reduction. Conversely, RF and WP elicited heightened physiological alertness (likely due to dense vegetation) but were subjectively perceived as having higher restorative potential due to biodiversity.

**Discussion:**

The study highlights a divergence between objective physiological recovery and subjective psychological preference. We conclude that urban planning must incorporate both ecological and sensory dimensions to maximize public health benefits across different green space types.

## 1 Introduction

Urban residents are increasingly recognizing the health benefits of exposure to nature, particularly its role in disease prevention (1–6). Urban green spaces contribute significantly to human health by providing essential ecosystem services, such as air purification, noise reduction, temperature regulation, and enhancing mental well-being through aesthetic value (7, 8). Recent studies emphasize the importance of multisensory experiences—visual, auditory, and olfactory cues—provided by these green spaces, which are essential for improving mental health and overall well-being (9–12). Additionally, exposure to nature is associated with stress reduction, mood enhancement, and improved cognitive function (1–3, 11, 12). These benefits greatly enhance the quality of life, foster social cohesion, and encourage physical activity within urban populations.

Despite the tremendous social and economic growth driven by rapid urbanization, this development has introduced significant challenges, such as air pollution, traffic congestion, noise, and social stressors, which negatively impact residents’ well-being, contributing to increased rates of stress, anxiety, and depression (11, 12). In response, urban green spaces have become a critical resource for city dwellers to reconnect with nature and mitigate these adverse health effects. These spaces provide essential opportunities for relaxation and rejuvenation, offering refuge from the hustle and bustle of urban life. However, due to the fast-paced urban lifestyle, residents are spending less time in green spaces, which has led researchers to focus on optimizing the health benefits that can be gained during short periods of exposure (13–15). As urban populations continue to grow, the need to understand and enhance the health-promoting potential of urban green spaces is more urgent than ever.

Studies on the effects of green spaces on health typically rely on physiological health indicators such as electroencephalogram (EEG), heart rate, and blood pressure (6, 16–18). It is important to note that these indicators measure acute “physiological responses” and “stress recovery” rather than “physiological health” per se, which refers to a long-term state. However, cumulative short-term restoration is a critical pathway contributing to long-term well-being. It is well-established that exposure to green spaces plays a crucial role in enhancing mental well-being, reducing stress, and promoting physical recovery (3, 11–13, 19). Current literature has begun to explore how specific variations in green space characteristics differentially impact health outcomes. For instance, recent empirical studies indicate that landscape characteristics and environmental types of urban greenways significantly affect physical and mental health restoration (20). Furthermore, specific attributes such as vegetation types and slope positions in urban forests have been identified as key factors influencing restorative effects (21). External factors also play a role; for example, different environmental types of urban green spaces have been shown to exert varying effects on public physiological and psychological health under different weather conditions (e.g., cloudy vs. sunny) (22–25).

To address the complexity of these health interactions, recent research has increasingly adopted the Physiological Health Effects (PHE) threshold model as a key analytical framework (11, 12). Unlike traditional linear models, the PHE model integrates the economic principle of diminishing marginal utility to quantify the non-linear dynamics of health restoration. Its primary advantage lies in its ability to identify specific critical points—namely the efficiency, benefit, and hazard thresholds—allowing for a precise evaluation of the optimal “dose” of nature required for maximum health returns. Recent progress in this field has successfully applied the PHE model to assess exposure to natural forests and outdoor environments, revealing that health benefits do not increase indefinitely but show diminishing returns as exposure duration and intensity increase (1, 11, 12, 26). Although short-term exposure to nature can quickly improve physical health, the long-term sustainability of these benefits tends to diminish over time (1, 27, 28). EEG has proven particularly useful in revealing immediate brain activity changes related to stress relief and cognitive enhancement triggered by green space exposure (6, 17, 18).

However, despite these methodological advancements, a significant gap remains in understanding whether the PHE threshold model is applicable to diverse types of urban green spaces. Much of the existing research has focused on urban parks and natural forests, yet it remains unclear whether these findings can be generalized across different types of urban green spaces with varying ecological characteristics (11, 12). Moreover, little attention has been given to the combined effects of demographic factors, meteorological conditions, and biodiversity indices on health outcomes. To address this gap, further research is needed to examine how various green space types interact with these factors, thereby offering a more nuanced understanding of their effects on urban residents’ health (20–23).

This study aims to explore the complex relationships between urban residents’ exposure to different types of green spaces and their acute physiological restoration outcomes. We conducted a scenario-based experiment using EEG devices to monitor physiological health responses, while also considering demographic factors, environmental conditions, and perceived biodiversity. Our study includes a range of green space types—remnant forests, wetland parks, and urban parks—to compare their respective health benefits and uncover the underlying mechanisms. The research addresses the following key scientific questions: (1) Is the PHE threshold model of health benefits from green space exposure applicable to different types of urban green spaces? (2) Do different types of green spaces exhibit distinct exposure-response patterns in terms of health outcomes? (3) How do health effects vary across green spaces with different levels of naturalness, ranging from more natural to more urbanized environments? By addressing these questions, the study will contribute to our understanding of how different types of urban green spaces impact human health and inform future urban planning strategies.

## 2 Materials and methods

### 2.1 Visual naturalness

To assess the level of visual naturalness in urban green spaces, we calculated the average percentage of visually natural elements based on multiple 180-degree panoramic photographs taken at each location. To ensure statistical robustness and capture the inherent heterogeneity of the environments, we selected 5–10 random sampling points within each green space type (Urban Parks, Forests, and Wetlands) rather than relying on a single viewpoint. This assessment combined computer vision techniques with manual evaluation to classify the proportion of natural and man-made features within each scene. First, we applied a semantic segmentation algorithm to analyze the scene captured in the view frame. Semantic segmentation is a technique that combines semantic and instance segmentation by identifying countable structures (such as trees and people) and uncountable structures (such as the sky), labeling each pixel to categorize the scene (29).

We used Fully Convolutional Networks (FCNs), a widely adopted method for semantic image segmentation, capable of identifying up to 150 site elements (29). In this study, spatial images from all sampling points were analyzed using a fully trained FCN model, with an average accuracy of over 90% (29, 30). Afterward, the results were manually reviewed and corrected to rectify any segmentation errors and to systematically aggregate the identified elements into the study’s target categories. Specifically, precise pixel-level adjustments were made to ensure that all landscape features were accurately assigned to either ‘natural’ or ‘man-made’ classes, prior to the exclusion of non-relevant elements (e.g., sky and pedestrians). The site features were then categorized into two groups: natural and man-made elements. Sky and human pixels were excluded from the analysis. The proportion of natural features was summed and averaged across all sampling points to calculate the overall visual naturalness of the green space.

### 2.2 Physiological health effect threshold model

This study utilizes the PHE threshold model to quantitatively evaluate the impact of urban green space exposure on human health. The model is constructed based on the dose–response relationship and the economic principle of diminishing marginal utility. It provides a theoretical framework to identify the non-linear dynamics between exposure duration (dose) and physiological health outcomes.

**Figure 1:**
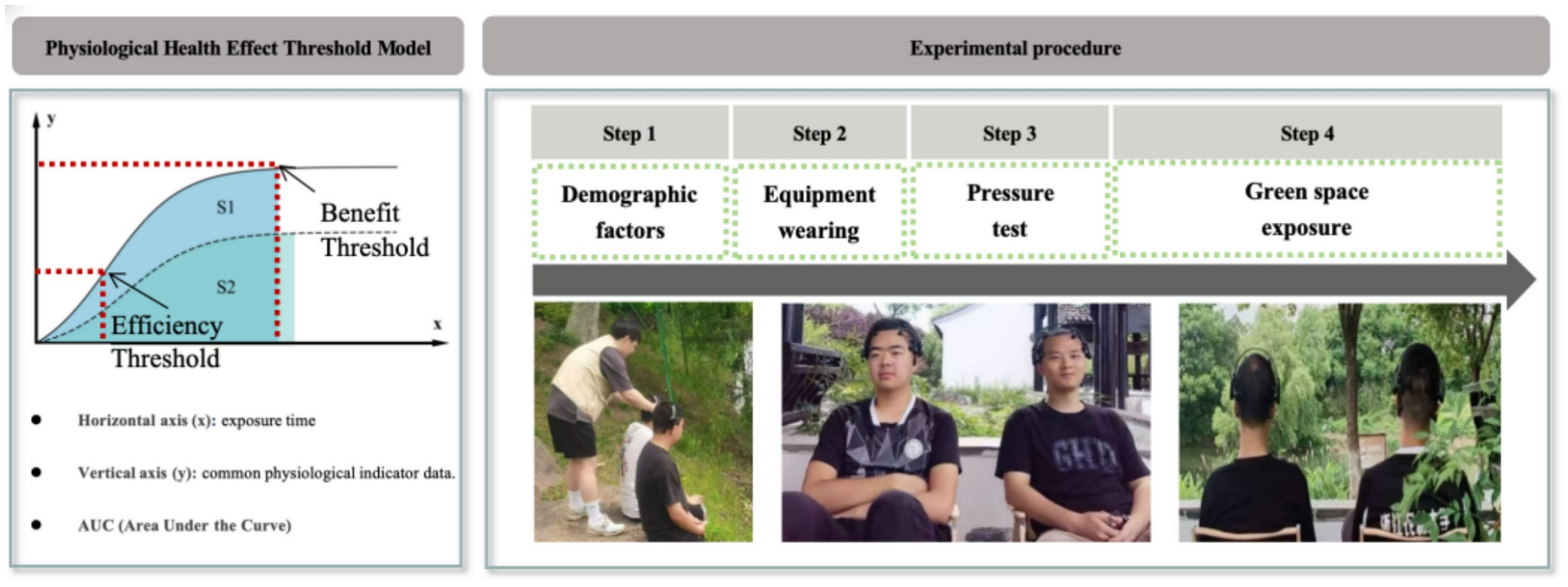
Overview of the physiological health effects (PHE) threshold model and the green space exposure experiment procedure.

In this framework (Figure 1), the *x*-axis represents the duration of exposure to green spaces (e.g., remnant forests, wetland parks, or urban parks), while the *y*-axis represents the standardized physiological health indicators (e.g., EEG). The curve illustrates how health benefits accumulate over time and eventually plateau. Based on this trajectory, the model identifies two critical thresholds: (1) Efficiency Threshold (ET): This point marks the onset of rapid recovery, where the marginal health benefit of additional exposure begins to increase significantly. (2) Benefit Threshold (BT): This point marks where the exposure benefits are maximized and begin to plateau. It indicates the optimal level of green space exposure for health improvement, beyond which additional exposure yields diminishing returns.

### 2.3 The definition and calculation of thresholds and AUC

To comprehensively evaluate the cumulative positive impact of green space exposure, we utilized the Area Under the Curve (AUC) method. AUC is widely used to evaluate dose–response relationships, particularly in studies exploring the link between natural exposure and health benefits (11, 12).

In this study, the AUC represents the total volume of physiological recovery accumulating over the exposure period. We calculated the AUC using the trapezoidal rule, which allows for the accurate quantification of cumulative effects for continuously differentiable functions within a specified interval. The calculation formula (1) and (2) are as follows:


$$\mathrm{AU}C_{1}=\int_{a}^{b}f_{1}(x)\mathrm{dx}=S_{1}$$ (1)

$$\mathrm{AU}C_{2}=\int_{a}^{b}f_{2}(x)\mathrm{dx}=S_{2}$$ (2)

Where *S* represents the accumulated health benefit (AUC), and the functions *f(x)* and *d(x)* represent the fitted dose–response curves for the respective physiological indicators over the exposure time *x*.

### 2.4 Case study

This study was conducted at the Xishanyang National City Wetland Park in China (30°N, 120°E). The experiment took place over 3 days in May 2024, from 8:30 a.m. to 5:30 p.m. each day. The study involved a total of 82 participants (detailed in Section 2.4.2). To ensure rigorous control over the experimental conditions, the study was conducted in multiple sessions.

**Figure 2:**
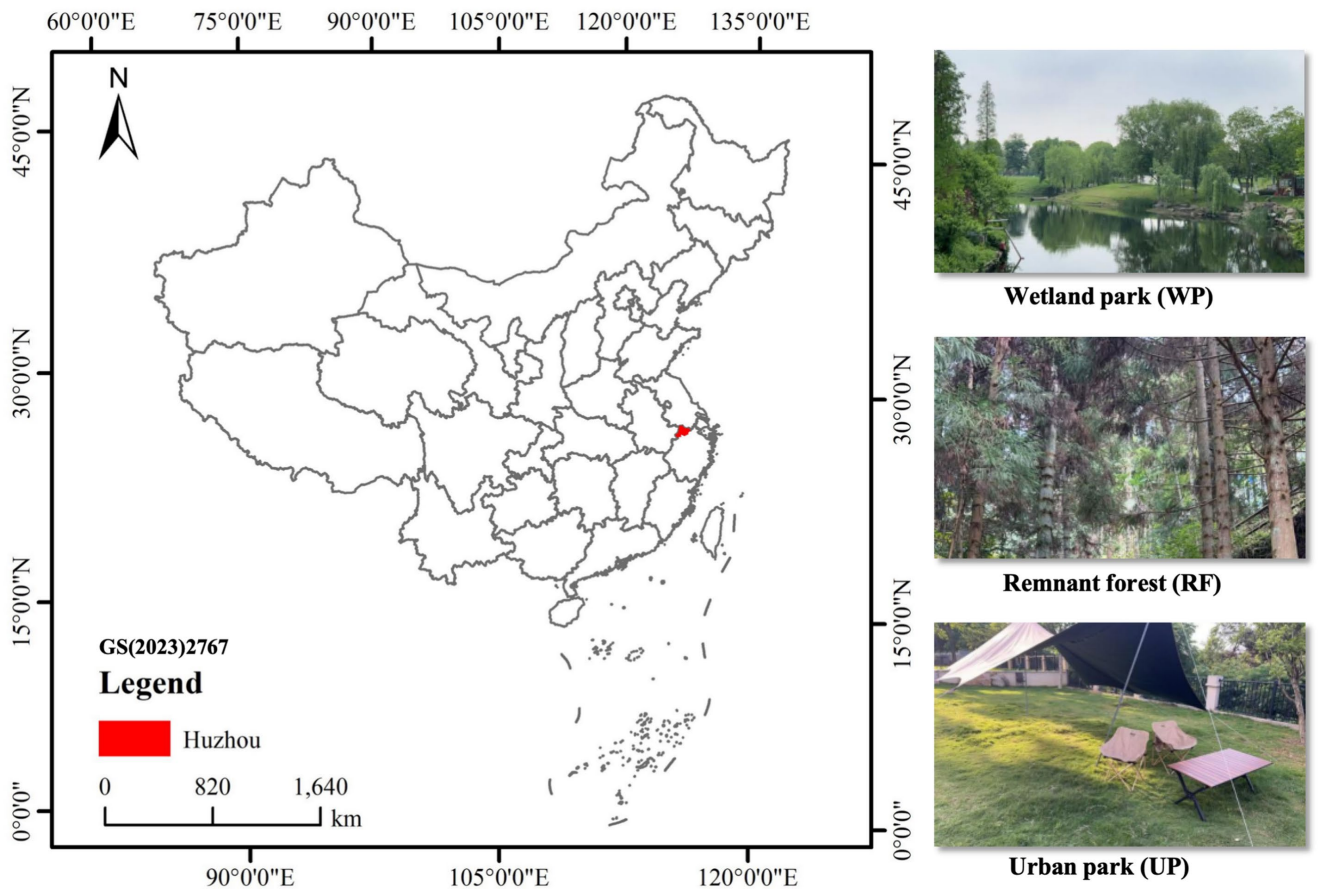
Map of the study area in China and photos illustrating the three green space types: remnant forest (RF), wetland park (WP), and urban park (UP).

In each session, participants were divided into two experimental conditions: the Open Eyes (OE) group and the Blindfold (BF) group. This quasi-experimental design exposed participants to different types of urban green spaces (UGS) along walking routes that included a park, a forest, and a wetland, providing three distinct natural environments for comparison (Figure 2). To assess the restorative effects, we used the Perceived Recovery Scale (PRS), which evaluates the restorative impact of visual stimuli. Participants were asked to undergo fatigue assessments before and after the exposure to minimize any potential “artificial” effects, ensuring the experiment reflected real-life conditions. The study aimed to quantitatively compare the physiological health effects of exposure to various UGS types on urban residents.

#### 2.4.1 Experimental procedure

Previous studies on the PHE of urban green space exposure have shown that both physical and mental health can improve rapidly after short-term exposure to green spaces. This study aims to evaluate the health threshold effects of various urban green space types (Figure 1). The experimental procedure is as follows:

Preparation: Participants first signed an informed consent form, followed by completing questionnaires related to demographics, perceived recovery, and their overall enjoyment of the soundscape.

Initial Setup: Each participant was equipped with an Emotiv EPOC X EEG headset. A 4-min stress test, involving complex numerical calculations paired with loud music, was performed to create a controlled stress environment.

Exposure Arrangement: To ensure the representativeness of the experimental sites, a comprehensive visual survey was conducted prior to the experiment, involving 5–10 sampling points per site to determine the average visual naturalness (as described in Section 2.1). Based on this analysis, a designated “docking station” that statistically represented the average landscape characteristics of each green space type was selected for the experiment.

During the formal experiment, participants underwent a 16-min stationary exposure session at each of the three environments: Urban Parks (UP), Remnant Forests (RF), and Wetland Parks (WP). To investigate the specific mechanisms of sensory recovery, participants were divided into two experimental conditions: (1) Open Eyes (OE) Group: Participants in this group were exposed to the full multisensory environment, integrating visual, auditory, and olfactory stimuli. (2) Blindfold (BF) Group: Participants wore standard opaque eye masks throughout the session to eliminate visual input. This group served as a comparative control to isolate the physiological health effects of non-visual sensory inputs (specifically auditory and olfactory cues) from visual naturalness. By comparing the OE and BF groups, we aimed to determine the extent to which visual perception contributes to the overall health restoration provided by urban green spaces.

During each session, participants remained seated at these representative “docking stations.” Data on biodiversity, meteorological conditions, and panoramic landscape photography capturing the participants’ full field of view were also recorded.

Repetition of Stress Tests and Baseline Standardization: To mitigate the variability in participants’ physiological states and minimize carry-over effects from previous sessions, a standardized stress test was administered before each of the three exposure sessions. While we acknowledge that individual stress responses vary, this procedure aimed to reset participants’ physiological baselines to a comparable state of cognitive fatigue and high arousal. During this phase, real-time EEG monitoring was employed to verify stress induction. Since Beta waves cannot be discerned from raw EEG signals, the experimenters utilized the real-time frequency spectrum display provided by the data acquisition software (Emotiv PRO). This interface performs instantaneous signal processing to visualize the power spectral density, allowing the researchers to observe the increase in relative Beta band activity (an indicator of alertness and stress) relative to the participant’s resting state. Only after the stress induction was confirmed effective—ensuring a valid starting point for measuring recovery—did the green space exposure session begin.

Duration: The entire experimental procedure lasted approximately 50 min, providing a controlled environment for assessing the physiological health effects of green space exposure under consistent stress induction conditions (Figure 1).

The EEG data were analyzed based on established correlations, including the relative alpha and beta indices, beta/alpha ratio, and the combined alpha + theta index. A higher relative alpha index is associated with relaxation, while a higher beta index indicates stress, anxiety, or preoccupation. The beta/alpha ratio reflects calmness, and the alpha + theta index is related to cognitive depth.

#### 2.4.2 Collection of demographic and environmental factors

Throughout the experiment, various environmental and human factors were measured both objectively and subjectively. Meteorological data, including temperature (°C), wind speed (m/s), relative humidity (%), atmospheric pressure (hPa), light intensity (lx), and carbon dioxide concentration (ppm), were recorded every 5 min.

The study involved 82 participants, ranging in age from 18 to 52 years, including students, visitors, and park staff. Before participation, everyone was informed about the experimental purpose and procedures, and all participants signed an informed consent form. Participants were also required to confirm that they had no history of physical or mental illness. The study adhered to ethical standards set by the Chinese Science Ethics Committee and followed the 1964 Declaration of Helsinki and its subsequent amendments, ensuring participant rights, privacy, and well-being were protected.

The on-site questionnaire collected data on participants’ individual characteristics, as well as their assessments of the landscape and acoustic environment. The Perceived Recovery Scale (PRS) (Supplementary Table S1) was used to evaluate the restorative effects of the green space exposure. The WHO-5 Well-being Index (Supplementary Table S2) was also used. Demographic data, including sex, age, height, weight, occupation, and general physical and mental health status (on a scale from 0 to 5, from “very poor” to “very good”), were also gathered. The questionnaire also assessed participants’ exposure habits, such as the frequency of green space exposure and their perceptions of site diversity, with scores ranging from 0 to 5, indicating a higher perceived diversity in the environment.

#### 2.4.3 Plant species richness assessment

To quantify the biodiversity of each experimental site, a field survey was conducted to assess plant species richness. A 10 m × 10 m quadrat (100 m^2^) was established, centered on the participant’s “docking station” to represent the immediate visual and ecological environment. Within each quadrat, a comprehensive inventory of all vascular plants was conducted, categorizing them into three vertical layers: arbor (trees), shrub, and herb layers. Species richness was calculated as the total count of distinct species identified within the sampling unit. This data was verified by botanical experts to ensure taxonomic accuracy.

### 2.5 Data analysis

The EEG signals were acquired using the Emotiv EPOC X wireless headset (Emotiv Systems, San Francisco, CA, United States). The device consists of 14 saline-soaked felt sensors located at AF3, F7, F3, FC5, T7, P7, O1, O2, P8, T8, FC6, F4, F8, and AF4, adhering to the international 10–20 system. Two references (CMS/DRL) were placed at the P3 and P4 positions. The sampling rate was set to 256 Hz (internally down-sampled to 128 Hz), with a bandwidth of 0.2–43 Hz. The impedance of all electrodes was monitored in real-time and maintained below 20 kΩ to ensure high signal quality.

Signal Preprocessing: Raw EEG data were exported and processed using MATLAB R2022b (MathWorks, Natick, MA, United States) and the EEGLAB toolbox. To remove noise and artifacts, the following preprocessing steps were applied: (1) Filtering: A high-pass filter at 0.16 Hz and a low-pass filter at 43 Hz were applied to remove baseline drift and high-frequency noise. A notch filter at 50 Hz was strictly applied to eliminate power line interference. (2) Artifact Removal: Eye blink and muscle movement artifacts were identified and corrected using Independent Component Analysis (ICA). Segments with voltage amplitudes exceeding ±100 μV were automatically rejected.

Feature Extraction: Fast Fourier Transform (FFT) was performed to convert the time-domain signals into the frequency domain (Power Spectral Density, PSD). The specific frequency bands were defined as follows: *θ* (Theta, 4–8 Hz), *α* (Alpha, 8–13 Hz), and *β* (Beta, 13–32 Hz). The relative power of each band was calculated as the ratio of the specific band’s power to the total power (4–32 Hz). Physiological indicators, including the Relative α index (relaxation), Relative β index (stress), and β/α ratio (alertness), were then computed for the “Stress” (0–4 min) and “Exposure” (4–20 min) phases. Data points were fitted every 30 s to generate trend curves. Specifically, the EEG data from all participants in each group were aggregated at each time point to construct the group-level dose–response trajectory.

## 3 Results

### 3.1 Demographic characteristics and environmental consistency

A total of 82 participants completed the experiment. The study population consisted of 45 males and 37 females. The participants included university students, park visitors, and staff members, ensuring a diverse representation. All participants reported good general physical and mental health status prior to the experiment.

Meteorological data monitored during the experiment (temperature, humidity, wind speed, and noise levels) were analyzed to ensure environmental consistency. The average temperature was 24 °C, and the relative humidity was 65.4%, and wind speed was 1.2 m/s. ANOVA results indicated no significant differences in meteorological conditions across the three experimental days (*p* > 0.05), ruling out weather as a confounding variable.

### 3.2 The visual naturalness and plant species richness

The visual naturalness of each location was assessed using a well-trained Fully Convolutional Network (FCN) model to identify site characteristics, specifically the percentage of natural elements in the environment. After manual inspection and re-classification, the results revealed that city parks (UP) had a significantly lower visual naturalness compared to forests (RF) and wetlands (WP). The visual naturalness scores were 85% for UP, 99% for RF, and 95% for WP.

For plant species richness, data were collected within a 100 m^2^ area around the experimental stopping points. The results showed that the wetlands had the highest plant species richness, followed by forests, with urban green spaces having the lowest richness.

### 3.3 Influence of exposure to UP, RF and WP on EEG indicators

**Figure 3:**
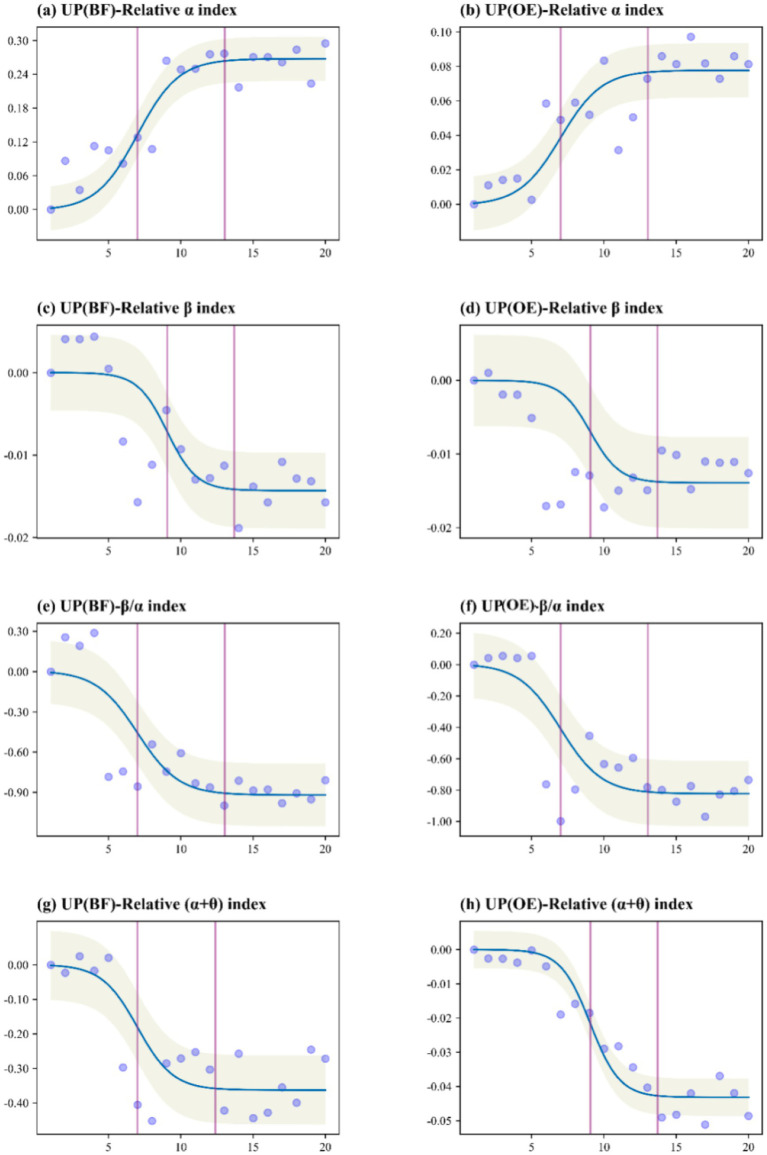
EEG measurement threshold curve for exposure to the urban park (UP) natural environment.

**Figure 4:**
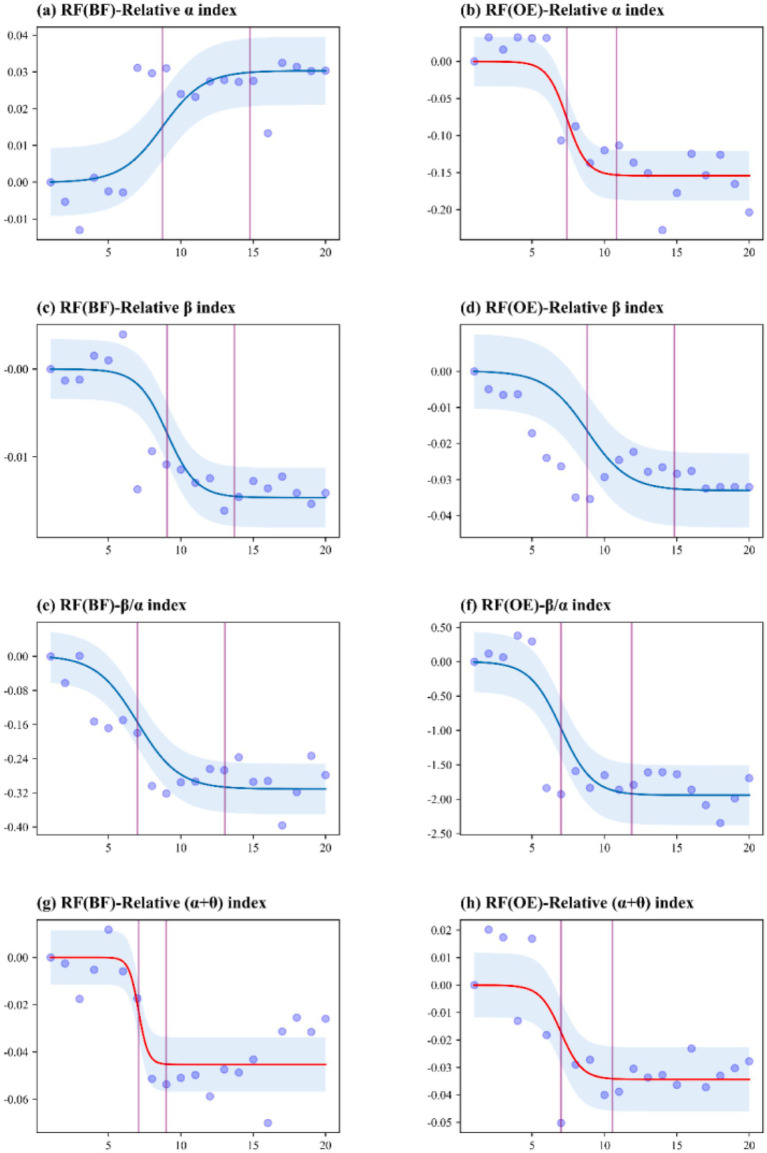
EEG measurement threshold curve for exposure to the remnant forest (RF) natural environment.

**Figure 5:**
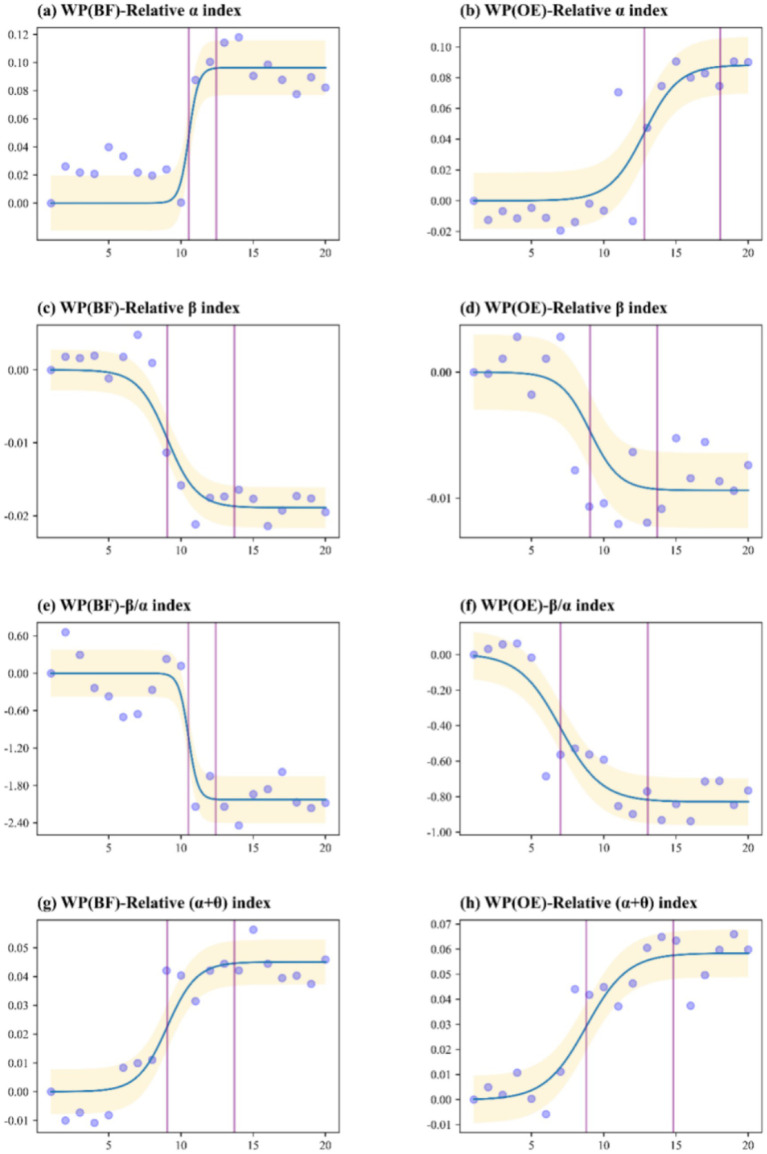
EEG measurement threshold curve for exposure to the wetland park (WP) natural environment.

Exposure to different urban green spaces (UP, RF, and WP) had significant effects on all selected EEG metrics (*p* < 0.05). To accurately interpret the temporal dynamics shown in Figures 3–5, it is essential to distinguish between the two experimental phases represented on the *x*-axis (0–20 min): (1) Stress Induction Phase (0–4 min): During this initial period, participants performed stress tasks. As shown in the figures, stress-related indicators (e.g., Relative β index) exhibited an upward trend or remained at a high level, confirming the successful induction of physiological stress prior to exposure. (2) Green Space Exposure Phase (4–20 min): This phase corresponds to the actual viewing of the green spaces. Our analysis of health restoration specifically focuses on this window.

Analyzing the Exposure Phase (4–20 min), participants in the OE group exposed to the UP environment exhibited significant improvements. Relaxation occurred rapidly after the onset of exposure (i.e., immediately after the 4-min mark), showing a more pronounced reduction in stress, as indicated by positive EEG changes (Figure 3). In comparison, the BF group also experienced relaxation and stress relief in the UP environment, but the temporal trajectory indicates that effects were slower and weaker than those observed in the OE group (Figure 3). Interestingly, neither the OE nor the BF group was able to engage in deep thinking while exposed to the urban park environment. This lack of effective deep thinking persisted throughout the exposure time, which is likely due to external factors such as noise, a fixed sitting posture for extended periods, and other environmental conditions.

Exposure to the RF environment, as indicated by the EEG results, shows that the forest environment contributes to an overall improvement in participants’ mental and physical health, promoting relaxation and stress relief (Figure 4). However, it is noteworthy that the relative *α* index and the relative (α + θ) index were negatively affected in the OE group over the course of the experiment (Figures 4b,h). These negative effects may be attributed to the fear and anxiety experienced by participants with open eyes in the natural forest, suggesting that visual exposure to dense forests may trigger complex psychological fluctuations over time rather than a linear recovery. In contrast, blindfolded participants in the RF environment appeared more relaxed. However, their heightened alertness and anxiety might have prevented them from achieving a state of deep thinking or full immersion in the experience. Overall, the OE group exhibited more significant physical health benefits than the BF group.

Regarding the temporal trends in the WP environment, the results revealed that both the OE and BF groups experienced a reduction in urban stress, fostering a relaxed and happy state throughout the exposure session (Figure 5). However, a negative effect was observed on the *β*/*α* ratio in the BF group (Figure 5e). This could be attributed to participants feeling panic and anxiety in the dark environment, leading to sustained heightened vigilance and preventing them from achieving a calm state.

In summary, when comparing the EEG indicators of the OE groups exposed to three natural environments—UP, RF, and WP (Table 1)—it is evident that all three environments contributed to improvements in participants’ psychological and physical health, promoting relaxation and reducing stress. Notably, the relative *α* index showed a negative effect during exposure to the RF environment compared to the other two green spaces (Table 1), which could be due to the panic experienced in the forest. Comparison of the three green spaces in terms of the relative *β*/*α* ratio revealed that all three environments helped reduce alertness, alleviate pressure, and foster inner peace (Table 1). AUC analysis also confirmed that all three environments contributed to better physical and mental health and helped ease urban stress. The relative (*α* + *θ*) index indicated that deep thinking was not effectively engaged in either the UP or RF environments (Table 1). This was attributed to the noise and artificial structures in the UP environment, which hindered deep thinking, while exposure to the RF environment led to fear and anxiety, further preventing participants from engaging in deep thought.

**Table 1:** Comparison of the efficiency threshold (ET), benefit threshold (BT), and area under the curve (AUC) for urban parks, remnant forests, and wetland parks.

EEG index	Urban Park			Remnant Forest			Wetland Park		
	ET (min)	BT (min)	AUC	ET (min)	BT (min)	AUC	ET (min)	BT (min)	AUC
Relative α	3	9	0.47	3	7	−0.53	9	14	0.47
Relative β	5	10	−0.06	5	11	−0.2	5	10	−0.04
β/α index	3	9	−4.94	3	8	−9.48	3	9	−4.98
Relative (α + θ)	5	10	0.2	3	7	−0.12	5	11	0.35

### 3.4 Psychological health effects

Subjective assessments corroborated the physiological EEG findings, showing significant psychological benefits. (1) Perceived Recovery Scale (PRS): Participants perceived the Remnant Forest (RF) and Wetland Park (WP) as significantly more restorative than the Urban Park (UP). The average PRS scores were 4.2 (RF) and 4.1 (WP), compared to 3.6 (UP) (*p* < 0.05), aligning with the higher visual naturalness of these sites. (2) WHO-5 Well-being Index: The WHO-5 scores significantly improved after exposure. The average score increased from 52.1 (Pre-exposure) to 68.4 (Post-exposure) (*p* < 0.01), confirming that short-term green space exposure effectively enhances immediate subjective well-being.

## 4 Discussion

### 4.1 The significance of the PHE threshold model in green space exposure

Exposure to green spaces has become a central focus in the integration of ecology and public health, shifting the focus from passive “downstream” health outcomes to active “upstream” health interventions (13, 31–35). By proposing the PHE threshold model, this study systematically assesses both the positive and negative effects of green space exposure on human health. The model provides a framework that not only quantifies the health benefits of green space exposure but also addresses potential adverse effects, such as the degradation of urban greenery, offering a more comprehensive understanding of the health impacts associated with green space.

The PHE threshold model plays a crucial role in guiding more scientifically informed green space exposure practices. By quantifying the PHE thresholds, the model helps maximize health benefits within a short exposure time, ensuring that residents gain the most from their time spent in green spaces (11, 12, 36, 37). Additionally, understanding the factors that influence these thresholds will assist urban planners and decision-makers in creating and maintaining effective green spaces. Overall, the model contributes to both theoretical and practical aspects of green space exposure research, providing valuable insights into the relationship between green space exposure (GE) and its associated health outcomes.

### 4.2 Evaluating the physical health effects in different green environments

The primary goal of this study was to assess the physiological health effects of different types of urban green spaces, including remnant forests, wetland parks, and urban parks. Our results confirm that the three green space types align well with the PHE threshold model, with distinct thresholds for efficiency, benefit, and harm. In line with previous research and supporting our hypothesis (33, 34), we found that green spaces with higher naturalness, such as forests and wetlands, produce stronger physiological health benefits compared to urban parks, which show lower restorative effects. The comparison of time to reach the threshold and AUC (Table 1) revealed that participants achieved the most significant physical health recovery in the RF environment, followed by the WP environment, with the UP environment showing the lowest recovery effect. This finding further supports the notion that more natural environments are more beneficial for urban residents, especially for weekend or leisure activities aimed at stress relief (38–40).

Furthermore, consistent with other studies (36, 37), our results suggest that “blue spaces” (water-related green areas) have the highest potential for physical and psycho-logical recovery (26, 41, 42). Notably, forest environments, with their higher biological recovery potential, outperform urban green spaces in promoting health (12). This indicates that different natural environments induce varying psychophysiological recovery mechanisms, emphasizing the importance of environmental complexity. According to Attention Restoration Theory, environments with richer biodiversity and more natural stimuli are more likely to attract attention effortlessly, leading to enhanced recovery (26, 40). Additionally, the microclimates created by biodiversity—such as the sounds of water, wind, and bird calls—contribute to faster and more significant health benefits, making these environments more conducive to physical and psychological rejuvenation (9, 11, 43).

### 4.3 Recovery potential from short natural exposures

While we hypothesized that more natural environments would exhibit greater recovery potential, an interesting finding emerged: regardless of environmental type, significant restorative effects were observed within a short period. EEG indices reached the efficiency threshold within approximately 9 min of exposure, and the benefit threshold was achieved within 14 min, particularly in forest environments (11–13). The slight variation in recovery times may be attributed to the relatively small sample size. Previous research on dose–response relationships has similarly indicated that even short exposure durations can promote physiological recovery, and that repeated short-term exposures may be more beneficial than prolonged single sessions (11, 12, 40, 41). Our findings extend this evidence by demonstrating measurable physiological changes across different types of natural environments during short exposures, thereby testing the applicability of the proposed PHE threshold model in quantifying both the positive and potential negative effects of green space exposure (Table 1).

This finding of rapid physiological restoration aligns with Ulrich’s Stress Reduction Theory (SRT), which posits that exposure to nature can trigger an immediate parasympathetic response to reduce arousal (41). Our results extend this theory by quantifying the specific time-dose required. Consistent with our observation of the 3–5 min efficiency threshold, previous laboratory studies have demonstrated that physiological stress markers (such as blood pressure and muscle tension) can drop significantly within just 3–4 min of viewing natural landscapes (7–9). Furthermore, recent multisensory experiments have confirmed that combining visual inputs with natural sounds (e.g., birdsong and water flow) and olfactory cues (e.g., phytoncides) significantly accelerates this recovery process compared to visual-only exposure (8, 42).

The superior performance of Remnant Forests (RF) and Wetland Parks (WP) in our study can be attributed to this multisensory synergy. Unlike urban parks, forests and wetlands provide a richer “soundscape” and distinct microclimatic benefits. For instance, wetlands generate higher concentrations of negative air ions and stable humidity, which have been shown to regulate autonomic nervous activity and enhance mood (26, 43). Similarly, the complex vegetation structure in forests not only offers high visual naturalness but also blocks urban noise and provides a sense of “being away,” facilitating deeper immersion (11, 12).

Therefore, our study validates the application of the PHE threshold model, confirming that while all green spaces reduce stress, the “dose” required to reach the benefit threshold varies by type. Short-term exposures (as brief as 10–15 min) in high-quality natural environments are sufficient to maximize physiological benefits, a finding that has significant implications for urban planning: specifically, that accessible “pocket” natural spaces may offer immediate relief for time-constrained urban residents (11, 12, 36, 44–46).

### 4.4 Limitations

This study has several limitations that should be addressed in future research. First, the short duration of the experiment introduced minor irregularities in the EEG data curves, making it challenging to precisely determine threshold points within the PHE model. Future studies should extend exposure durations and incorporate additional physiological indicators to enhance model accuracy and robustness. Second, controlling for unexpected outdoor disturbances was inherently limited; however, such variations represent an authentic aspect of natural exposure and contribute to the ecological validity of the findings. Importantly, no major external disturbances were reported by researchers or participants during the trials.

Furthermore, it is important to note the limited range of visual naturalness represented in the selected experimental sites. The three green space types analyzed—Urban Parks (85%), Wetland Parks (95%), and Remnant Forests (99%)—all possess relatively high levels of visual naturalness. The absence of a “low-naturalness” control group (e.g., built environments or gray spaces with minimal vegetation) restricts our ability to observe the physiological responses at the lower end of the environmental spectrum. Consequently, the threshold model derived in this study may primarily reflect the dynamics within high-quality green spaces. Future research should aim to include a broader gradient of settings, ranging from heavily urbanized areas to pristine nature, to fully characterize the dose–response relationship and identify health thresholds across diverse environmental characteristics. Finally, while this study successfully established the PHE threshold model using EEG data, we acknowledge that it focused on a single physiological dimension. Future research will expand the validation of the PHE model by incorporating additional physiological indicators, such as Heart Rate Variability (HRV) and Blood Pressure (BP). Integrating these metrics will provide a more comprehensive multidimensional perspective on the health restoration thresholds of urban green spaces. Integrating these factors will help refine the PHE threshold model and provide stronger empirical evidence for planning forest and wetland environments that optimize both ecological sustainability and public health benefits.

## 5 Conclusion

This study applied an advanced Physiological Health Effects (PHE) threshold model to evaluate the impacts of green space exposure on human health. By utilizing EEG data, we established distinct efficiency, benefit, and hazard thresholds, which enabled the observation of significant physiological changes among participants exposed to different types of natural environments during relatively short exposure periods.

The study further compared the physiological restorative benefits across various types of urban green spaces, revealing a distinct divergence between physiological and psychological restorative outcomes: (1) Physiological Health Effects: UP exhibited stronger and more immediate physiological restoration effects (e.g., rapid Alpha wave increase) compared to RF and WP. This suggests that accessible, well-maintained urban parks may offer a more stable environment for rapid physiological stress recovery, likely due to a greater sense of safety and lower cognitive demand. (2) Psychological Health Effects: In contrast, RF and WP contributed more prominently to subjective psychological restoration. Despite the physiological “alertness” observed in these environments, participants consciously rated them as having higher restorative quality (PRS scores) due to their superior visual naturalness and biodiversity.

The results suggest that urban parks, although characterized by lower levels of visual naturalness than forests and wetlands, may still provide substantial acute physiological restoration benefits due to their greater accessibility, recreational functionality, and social engagement opportunities. In contrast, while more natural environments (such as forests and wetlands) offer high scenic quality, their restorative efficacy in this study appeared to be moderated by participants’ psychological responses (e.g., alertness or anxiety), highlighting the need to balance ecological naturalness with a sense of security in urban green space design.

Future research should expand participant diversity and integrate additional environmental parameters—specifically measuring auditory soundscapes and olfactory variables—to fully understand the multisensory mechanisms of health restoration. Moreover, exploring how different demographic groups interact with various types of green spaces will support the development of personalized exposure strategies and evidence-based urban design guidelines. Ultimately, this research provides valuable insights for urban planners and policymakers to design green spaces that not only maximize physiological restoration and psychological well-being but also mitigate the pressures of rapidly urbanizing environments.

## Data Availability

The original contributions presented in the study are included in the article/Supplementary material, further inquiries can be directed to the corresponding authors.
