# Supplementary material for: Physiological restorative benefits of different types of urban green spaces: a threshold model approach
Source: Front Public Health. 2026 Feb 6;14:1735199. doi: 10.3389/fpubh.2026.1735199 (PMC12921484; doi:10.3389/fpubh.2026.1735199)
Supplement: Supplementary file 1 [file Table_1.DOCX]

Supplementary Material

Table S1. Descriptive statistics of Perceptual Recovery Scale (PRS) scores

| number | Being Away Mean±SD | Fascination Mean±SD | Coherence Mean±SD | Compatibility Mean±SD | Total score Mean±SD |
| --- | --- | --- | --- | --- | --- |
| 82 | 4.18±2.12 | 3.02±2.21 | 3.52±1.98 | 3.04±1.51 | 3.34±1.39 |

**Note:** The Perceptual Recovery Scale (PRS) was employed to assess participant responses. The PRS is composed of five sections, totaling 23 questions, each scored on a 0-7 scale. The sections are organized as follows: the first question assesses overall evaluation; questions 2 through 6 measure the sense of being away ('Distance'); questions 7 through 14 explore 'Charm'; questions 15 through 18 assess 'Compatibility'; and questions 19 through 23 gauge 'Consistency'. This structure allows for a comprehensive evaluation of the restorative qualities of the environments under study.

Table S2. World Health Organization Five Physical and Mental Health Indicators (WHO-5)

| In the last two weeks | All the time | Most of the time | More than half the time | Less than half the time | A fraction of time | none |
| --- | --- | --- | --- | --- | --- | --- |
| 1. I feel happy and happy | 5 | 4 | 3 | 2 | 1 | 0 |
| 2. I feel peaceful and relaxed | 5 | 4 | 3 | 2 | 1 | 0 |
| 3. I feel alive and energetic | 5 | 4 | 3 | 2 | 1 | 0 |
| 4. I wake up feeling fresh and well-rested | 5 | 4 | 3 | 2 | 1 | 0 |
| 5. My everyday life is full of interesting things | 5 | 4 | 3 | 2 | 1 | 0 |
